# Model curriculum of the German society for Rheumatology for advanced training in the discipline internal medicine and rheumatology. English version

**DOI:** 10.1007/s00393-021-01080-6

**Published:** 2021-10-04

**Authors:** Alexander Pfeil, Martin Krusche, Diana Vossen, Michael N. Berliner, Gernot Keyßer, Andreas Krause, Hanns-Martin Lorenz, Bernhard Manger, Florian Schuch, Christof Specker, Jürgen Wollenhaupt, Xenofon Baraliakos, Martin Fleck, Fabian Proft

**Affiliations:** 1 Department of Internal Medicine III, University Hospital Jena, Am Klinikum 1, 07747 Jena, Germany; 2 Department of Rheumatology and Clinical Immunology, Charité University Medicine, Berlin, Germany; 3 Rheinisches Rheumazentrum Meerbusch-Lank, St. Elisabeth Hospital, Meerbusch, Germany; 4 Rheumatology and Geriatrics, Helios Klinikum Berlin-Buch, Berlin, Germany; 5 Department of Internal Medicine, Clinic for Internal Medicine II, University Hospital Halle, Halle (Saale), Germany; 6 Department of Internal Medicine, Division of Rheumatology, Clinical Immunology and Osteology, Immanuel Hospital Berlin, Berlin, Germany; 7 Division of Rheumatology, Department of Medicine V, University Hospital Heidelberg, Heidelberg, Germany; 8 Medical Clinic 3, Rheumatology and Immunology, University Hospital Erlangen, Friedrich-Alexander University Erlangen-Nuremberg, Erlangen, Germany; 9 Joint practice Internal Medicine Rheumatology—Nephrology, Erlangen, Germany; 10 Clinic for Rheumatology and Clinical Immunology, Evangelisches Krankenhaus Kliniken Essen-Mitte, Essen, Germany; 11 Rheumatology at Struenseehaus, Hamburg, Germany; 12 Rheumazentrum Ruhrgebiet, Ruhr University Bochum, Herne, Germany; 13 Clinic and Polyclinic for Internal Medicine I, University Hospital Regensburg, Regenburg, Germany; 14 Clinic and Polyclinic for Rheumatology/Clinical Immunology, Asklepios Klinikum Bad Abbach, Bad Abbach, Germany; 15 Department of Gastroenterology, Infectiology and Rheumatology (including Nutrition Medicine) , Charité – Universitätsmedizin Berlin, corporate member of Freie Universität Berlin and Humboldt-Universität zu Berlin, Berlin, Germany

**Keywords:** Model training regulations, Core competences, Residency, Further training section, Training institution, Musterweiterbildungsordnung, Kernkompetenzen, Facharztweiterbildung, Ausbildungsabschnitte, Weiterbildungsort

## Introduction

With the implementation of the revision of the model training regulations for German physicians (*Musterweiterbildungsverordnung*, MWBO) in 2018, the area of each field in internal medicine was redefined and a restructuring of the training period for specialists in internal medicine and rheumatology took place [2].

The minimum training time requires 72 months, divided into two parts of 36 months each, which include both the common contents of the training as a specialist in internal medicine (“basic training”) as well as the specific contents of the specialist training in internal medicine and rheumatology.

The basic training period in “internal medicine” requires acquisition of at least two specialist competences which do not belong to the specializing field. This training consists of 24 months and is complemented by 6 months of training in both an emergency department and an intensive care unit. The specialized training period in internal medicine and rheumatology also extends over 36 months, of which at least 24 months are completed in inpatient rheumatologic care as defined by the MWBO [2]. Exemptions from this regulation were introduced by individual State Medical Chambers (*Landesärztekammern*) to allow for a longer period of specialized training in outpatient rheumatologic care.

Based on the MWBO of 2018, a model curriculum was developed by the Commission for Education and Training of the German Society of Rheumatology (*Deutsche Gesellschaft für Rheumatologie*, DGRh) on behalf of the board of the DGRh with regard to core competences in specialized training in internal medicine and rheumatology. This model curriculum focuses on advanced training competences which should be acquired within the 36 months of specialized training in internal medicine and rheumatology and does not include competences relevant to the general part of internal medicine training (basic training).

In this review article, the model curriculum of the DGRh for specialized training in internal medicine and rheumatology is presented.

## The model training regulations (MWBO)

The MWBO 2018 provides “cognitive and methodological competences” (knowledge) and “action competences” (experience and skills) for the specialty of internal medicine and rheumatology [1, 2], which includes the recognition, diagnosis, differential diagnosis, treatment, long-term care, and rehabilitation of inflammatory rheumatic joint diseases, inflammatory or immunologic systemic diseases (especially connective tissue diseases and vasculitides), auto-inflammatory syndromes, immunodeficiencies, and their associated comorbidities [2]. Based on the MWBO 2018, the model curriculum was developed by the Commission of Education and Training of the DGRh.

## The model curriculum

The aim of the model curriculum is to establish a guideline for trainers and trainees in rheumatology, in which the training competencies of the 36-month training period in the specialty of internal medicine and rheumatology based on the MWBO 2018 are provided in a structured and synchronized manner [2]. The model curriculum is also intended to serve as a basis for all postgraduate trainers in the development of their own rheumatology training plan, which must be submitted to the State Medical Chambers with the new application for training authorization, as is mandatory with the implementation of the MWBO.

The defined examination and treatment procedures listed in the MWBO 2018 serve as the basis for this model curriculum [2]. The aim of the model curriculum is the structured learning of physical examination, diagnosis, differential diagnosis, treatment algorithms, long-term care, and rehabilitation of inflammatory rheumatic joint diseases, inflammatory or immunologic systemic diseases (especially connective tissue diseases, vasculitides, and auto-inflammatory syndromes), and their associated comorbidities.

## Structure of the model curriculum

The competences to be provided and acquired were divided into 3 years of training. These competences are minimum requirements, which should be adapted to the respective training institution, so that corresponding activities or knowledge can be learned or performed at an earlier (but also later) point in time. Of course, a more in-depth teaching of competences beyond the present model curriculum is possible and desirable and can be included accordingly in the individual specialized training plan of the training institution. The structure of the specialized training competences as demonstrated here was developed by the Commission of Education and Training and consented by the board of the DGRh and is intended to enable the acquisition of competences to be coordinated over time. In addition, variable training content is also included, which is not assigned to a training section and can be acquired according to the level of knowledge in the first to third year of training.

The acquisition of competences follows the structure of the curriculum with regard to learning of leading symptoms; examination; laboratory, radiology, rheumatologic diagnostics; physical therapy; and therapeutic measures. In addition, interventions (e.g., joint aspiration) are dedicated to the second and third year of training (for details, see Table 1).

**Table 1:** Minimum requirements for the acquisition of competences, whereby the activities and knowledge can be learned or performed at an earlier or later point in time than indicated

–	Acquisition of competences
1st year	*Leading symptom:* Learning of a targeted rheumatologic medical history with focus on the leading symptom and possible underlying inflammatory rheumatic diseases
	*Examination: *Learning of a targeted rheumatologic physical examination including potential systemic involvement of inflammatory rheumatic diseases
	*Laboratory:* Learning of a targeted indication as well as introduction to methodology, performance, and classification of laboratory tests and immunologic parameters in inflammatory rheumatic clinical pictures
	*Radiology:* Learning of the specific indication of radiologic examinations as well as the classification of the findings in inflammatory rheumatic clinical pictures
	*Rheumatologic diagnostics:* Introduction to specific rheumatologic/immunologic diagnostics regarding arthro- and vascular sonography, capillary microscopy, analysis of synovial fluid as well as rheumatologic/immunologic laboratory diagnostics including autoantibodies and immunogenetic testing under close supervision
	*Physical therapy:* Knowledge of physical, physiotherapeutic, and ergotherapeutic treatment principles in specific inflammatory rheumatic diseases and degenerative joint diseases and spinal diseases
	*Treatment:* Knowledge of creating a medication plan for the treatment of inflammatory rheumatic diseases and degenerative joint diseases Learning to recognize and deal with potential treatment-associated adverse events Independent prescription of medical treatment as well as medical supplies on the basis of the prescription for the treatment of inflammatory rheumatic diseases and degenerative joint diseases Learning of interdisciplinary indications for surgical, radiotherapeutic, and nuclear medicine treatment procedures Co-managing patients with inflammatory rheumatic diseases and degenerative joint diseases under supervision Creating structured rheumatologic reports/doctor’s letters under supervision
2nd year	*Leading symptom/diagnosis/differential diagnosis:* Advanced targeted rheumatologic medical history for differential diagnostic classification of symptoms described
	*Examination:* Advanced rheumatologic physical examination including possible systemic involvement of inflammatory rheumatic diseases Independent managing of patients with inflammatory rheumatic diseases and degenerative joint diseases under supervision
	*Radiology:* Advanced knowledge of the evaluation of radiologic procedures for the diagnosis of inflammatory rheumatic and degenerative joint diseases as well as possible treatment-associated adverse events and the differential diagnostic classification of these findings in inflammatory rheumatic clinical pictures
	*Rheumatologic diagnostics:* Independently perform targeted rheumatologic/immunologic diagnostic procedures using arthrosonography, vascular sonography, capillary microscopy, synovial fluid analysis, and rheumatologic/immunologic laboratory diagnostics including autoantibodies, cellular immunity, and immunogenetic testing under supervision
	*Interventions:* Introduction to invasive diagnostic and therapeutic procedures (such as joint puncture, injection therapies, and/or lip gland and skin biopsies) under close supervision
	*Treatment*: Independent creation of a drug therapy plan treating inflammatory rheumatic diseases and degenerative joint diseases including patient education under supervision
3rd year	*Medical history, diagnostics, treatment:* Independent management of patients with inflammatory rheumatic diseases and degenerative joint diseases with the possibility of consultation if required Acquisition of basic knowledge in physical and rehabilitative medicine as well as issues of rheumatologic assessment Acquisition knowledge of transitioning patients with pediatric rheumatologic conditions. Acquisition of basic knowledge for planning and managing pregnancies in female patients with inflammatory rheumatic diseases Acquisition of basic knowledge for the diagnosis and treatment of primary and secondary immunodeficiencies
	*Radiology:* Evaluation of radiologic procedures for the diagnosis of inflammatory rheumatic and degenerative joint diseases as well as possible treatment-associated adverse events and the differential diagnostic classification of these findings in inflammatory rheumatic clinical pictures
	*Rheumatologic diagnostics: *Independent performance of targeted rheumatologic/immunologic diagnostics by means of arthrosonography, vascular sonography (including duplex sonography for acute diagnosis of vasculitides), ultrasound of the salivary glands, capillary microscopy, analysis of synovial fluid, and rheumatologic/immunologic laboratory diagnostics including autoantibodies and immunogenetic testing with the possibility of consultation if needed, as well as learning quality assurance according to RiLiBÄK guidelines
	*Interventions: *Performing invasive diagnostic and therapeutic procedures (such as joint puncture and injection therapies and others) under supervision

Variable contents, which are not assigned to any further training section, are osteology, training programs, and issues of radiation safety (see Table 2).

**Table 2:** Further education contents with a variable assignment to the further education section

–	Acquisition of competences
Variable time assignment	*Osteology:* Diagnosis and conservative treatment of osteoporosis and osteomalacia or osteologic disease patterns Indication, performance, and evaluation of DXA bone mineral density measurement
	*Training programs*: To become acquainted with and learn structured patient education programs for rheumatic and musculoskeletal diseases
	*Radiation protection:* Basics of radiation biology and radiation physics in the use of ionizing radiation in humans Basics of patient and staff radiation protection including staff supervision and constructional and equipment-based radiation protection

## Adaptation of the model curriculum

The model curriculum reflects the minimum requirements of the MWBO. In this context, it is not to be regarded as an optimal and binding training option for every rheumatology training center in Germany, but rather provides the framework for successful training as a specialist in internal medicine and rheumatology. Adaptation of the curriculum to the respective situation of the specialized training location is possible or may be necessary at any time. This includes changes in the chronologic sequence of the competence transfer, a more in-depth training in individual aspects, and addition of further training content.

For a continuous development of the curriculum, the Commission for Education and Training asks particularly for feedback from the trainers and trainees regarding the content, topics, and structure of the curriculum. Thus, the curriculum is to be seen as a “work in progress,” which is why comments or suggestions for improvement to the commission are explicitly desired.

In summary, the model curriculum is intended to ensure standardized and structured training and continuing education for specialists in internal medicine and rheumatology.

## Conclusion for clinical practice

- The model curriculum enables the standardized teaching of core competences as part of specialist training in internal medicine and rheumatology.
- The core competences to be acquired and the variably assignable training contents are provided in three training sections over 36 months.
- An adaptation of the curriculum to the requirements of the respective institutions of specialized training is reasonable or may be necessary.

## Abbreviations

DGRh: German Society for Rheumatology
MWBO: Model training regulations for German physicians

## Declarations

For this article no studies with human participants or animals were performed by any of the authors. All studies performed were in accordance with the ethical standards indicated in each case.
